# Combined 3 Tesla MRI Biomarkers Improve the Differentiation between Benign vs Malignant Single Ring Enhancing Brain Masses

**DOI:** 10.1371/journal.pone.0159047

**Published:** 2016-07-13

**Authors:** Simone Salice, Roberto Esposito, Domenico Ciavardelli, Stefano delli Pizzi, Rossella di Bastiano, Armando Tartaro

**Affiliations:** 1 Department of Neurosciences, Imaging and Clinical Sciences, University “G. d’Annunzio” of Chieti-Pescara, Chieti, Italy; 2 AO Ospedali Riuniti Marche Nord, Pesaro, Italy; 3 School of Human and Social Science, “Kore” University of Enna, Enna, Italy; 4 Molecular Neurology Unit, Center of Excellence on Aging and Translational Medicine (Ce.S.I.-MeT), University “G. d’Annunzio” of Chieti-Pescara, Chieti, Italy; John Hopkins School of Medicine, UNITED STATES

## Abstract

**Purpose:**

To evaluate whether the combination of imaging biomarkers obtained by means of different 3 Tesla (3T) Magnetic Resonance Imaging (MRI) advanced techniques can improve the diagnostic accuracy in the differentiation between benign and malignant single ring-enhancing brain masses.

**Materials and Methods:**

14 patients presenting at conventional 3T MRI single brain mass with similar appearance as regard ring enhancement, presence of peri-lesional edema and absence of hemorrhage signs were included in the study. All lesions were histologically proven: 5 pyogenic abscesses, 6 glioblastomas, and 3 metastases. MRI was performed at 3 Tesla and included Diffusion Weighted Imaging (DWI), Dynamic Susceptibility Contrast -Perfusion Weighted Imaging (DSC-PWI), Magnetic Resonance Spectroscopy (MRS), and Diffusion Tensor Imaging (DTI). Imaging biomarkers derived by those advanced techniques [Cerebral Blood Flow (CBF), relative Cerebral Blood Volume (rCBV), relative Main Transit Time (rMTT), Choline (Cho), Creatine (Cr), Succinate, N-Acetyl Aspartate (NAA), Lactate (Lac), Lipids, relative Apparent Diffusion Coefficient (rADC), and Fractional Anisotropy (FA)] were detected by two experienced neuroradiologists in joint session in 4 areas: Internal Cavity (IC), Ring Enhancement (RE), Peri-Lesional edema (PL), and Contralateral Normal Appearing White Matter (CNAWM). Significant differences between benign (n = 5) and malignant (n = 9) ring enhancing lesions were tested with Mann-Withney U test. The diagnostic accuracy of MRI biomarkers taken alone and MRI biomarkers ratios were tested with Receiver Operating Characteristic (ROC) analysis with an Area Under the Curve (AUC) ≥ 0.9 indicating a very good diagnostic accuracy of the variable.

**Results:**

Five MRI biomarker ratios achieved excellent accuracy: IC-rADC/PL-NAA (AUC = 1), IC-rADC/IC-FA (AUC = 0.978), RE-rCBV/RE-FA (AUC = 0.933), IC-rADC/RE-FA (AUC = 0.911), and IC-rADC/PL-FA (AUC = 0.911). Only IC-rADC achieved a very good diagnostic accuracy (AUC = 0.909) among MRI biomarkers taken alone.

**Conclusion:**

Although the major limitation of the study was the small sample size, preliminary results seem to suggest that combination of multiple 3T MRI biomarkers is a feasible approach to MRI biomarkers in order to improve diagnostic accuracy in the differentiation between benign and malignant single ring enhancing brain masses. Further studies in larger cohorts are needed to reach definitive conclusions.

## Introduction

Single ring-enhancing brain masses include benign and malignant lesions of different aetiology. Imaging differentiation among them is crucial for treatment planning and prognosis estimation.

Single pyogenic abscesses are the most frequent “benign” lesions showing ring-enhancement at MRI [1]. Surgical drainage and administration of high doses of intravenous antibiotics ensure a full recovery in 70% of patients and a 10% fatality rate [2, 3].

Conversely, the most frequent “malignant” masses presenting similar imaging findings are metastases and glioblastomas (GBM) [4, 5]. Chemotherapy, surgery, whole brain radiation therapy, and stereotactic radiosurgery [6, 7] are therapeutic options for metastases treatment, whose median survival varies from a few months to less than two years on the basis of numerous demographic and clinical prognostic factors [8]. Despite the proven benefit of surgical resection, radiation therapy and temozolomide chemotherapy, GBM prognosis remains very poor [9].

Discrimination of these entities on the basis of conventional MRI findings other than contrast enhancement and solitary presentation is often possible. Typically, pyogenic abscesses have a smooth inner margin of the ring and a hypointense signal of the rim on T2-weighted images [10], while malignant lesions have irregular margins of the ring and lack a dark rim on T2-weighted images. When those findings cannot be clearly identified, the final diagnosis remains uncertain.

The Biomarkers Definitions Working Group (BDWG) of the National Institutes of Health defines a biomarker as “a characteristic that is objectively measured and evaluated as an indicator of normal biological processes, pathogenic processes, or pharmacologic responses to a therapeutic intervention” [11].

Nowadays, MRI is no longer considered just a qualitative diagnostic imaging method but also a quantitative tool.

Advanced MRI technique allow the quantification of metabolite rates, perfusion parameters and water diffusivity indices, opening new scientific applications with a shift from qualitative imaging to quantitative imaging [12, 13]

Diffusion Weighted Imaging (DWI), Diffusion Tensor Imaging (DTI), Magnetic Resonance Spectroscopy (MRS), and Perfusion Weighted Imaging (PWI) provide quantitative, reliable and reproducible information about microvascularity, neoangiogenesis, metabolism, necrosis and cellularity of brain masses [14]. Parameters obtained by means of these advanced MRI techniques can be considered as “biomarkers” to all effects.

Growing efforts have been addressed to define MRI biomarkers that can improve the differentiation between benign and malignant ring enhancing brain masses. Apparent Diffusion Coefficient (ADC), a biomarker derived from DWI, and Fractional Anisotropy (FA), a biomarker derived from DTI, are respectively significantly lower and higher in the central cavity of pyogenic abscesses than in the central cavity of necrotic tumours [15–19]; aminoacids, biomarkers derived from MRS, are usually revealed only in the central cavity of pyogenic abscesses and not in the central cavity of malignant tumors [17]; Cerebral Blood Volume (CBV), a biomarker derived from PWI, if measured in ring enhancement and perilesional edema, is generally higher in malignant lesions than in pyogenic abscesses [20, 21].

Biomarkers combination is a commonly used approach in many scientific fields in order to allow more accurate diagnostic judgment [22, 23, 24].

Nowadays, in none of the MRI studies focused on the discrimination of ring enhancing brain masses, biomarkers from different advanced MRI techniques have been combined each other. As in other medical research fields [25], we hypothesize that microstructural heterogeneity of a lesion can be described in a more comprehensive way by combining information from different MRI biomarkers.

In this study, we proposed to combine, by both univariate and multivariate approaches, each MRI biomarkers with the other ones in order to further improve the diagnostic accuracy of MRI advanced techniques, for the differentiation between benign and malignant single ring-enhancing brain masses.

## Materials and Methods

### Patients

This study was performed retrospectively. All data collected for clinical purpose were anonymous and the readers were the ones who performed all clinical examinations.

The study was approved by the Ethics Committee of the University “G. d’Annunzio” of Chieti—Pescara (Chieti, Italy)[26]. All patients or caregivers were aware that their data will be used for possible research purpose other than clinical diagnosis and they signed an informed consent.

**Fig 1:**
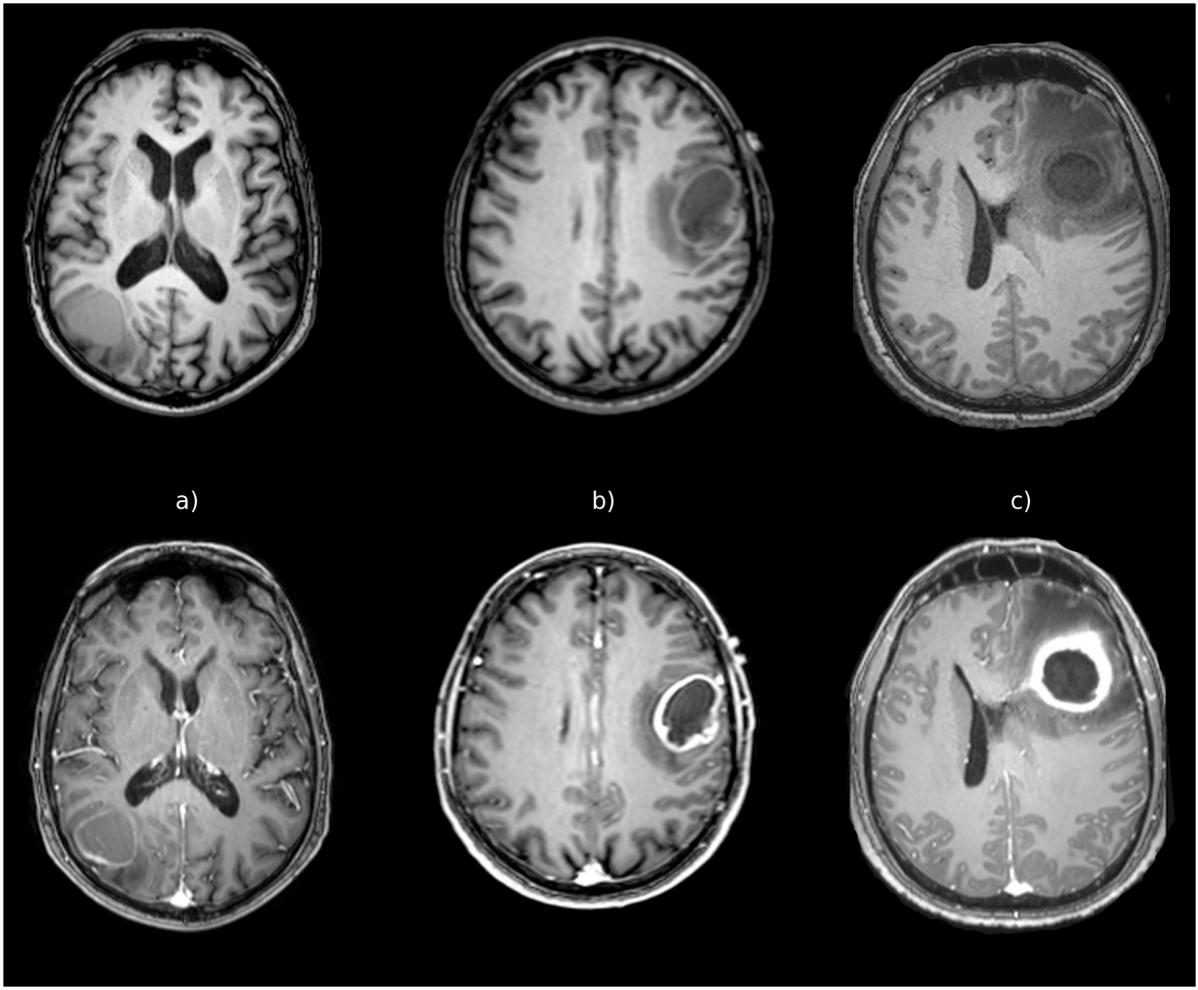
3D TFE T1-weighted sequence in the axial plane before and after contrast agent administration. Single ring-enhancing brain masses with surrounding edema. Histology proved in A. GBM, in B. metastasis, and in C. abscess.

The study included fourteen patients (11 men and 3 women; aged 33–78 years; mean age 62 years) presenting a single brain mass indistinguishable at conventional MRI and characterized by ring enhancement (Fig 1), peri-lesional edema, and absence of haemorrhage signs. None of the patients underwent surgery or biopsy before the MRI scan.

All lesions were histologically proven. 5 lesions were benign and all of them were pyogenic abscesses. 9 lesions were malignant and they included 6 GBMs, and 3 metastases.

All patient underwent the same advanced 3T MRI scan protocol.

MRI data were obtained with a 3T scanner (Philips Achieva X Series, Philips Medical System, Best, Netherlands) using a SENSE (Sensitivity Encoding) dedicated eight-channel head coil.

All patients underwent conventional and advanced MR techniques.

### Conventional MRI

Imaging protocol of conventional MRI included a 3D turbo field echo (TFE) T1-weighted sequence acquired on the sagittal plane [7.6 ms TR, 3.7 ms TE, 256x256 matrix, FOV 250x250x160, scan time of 1 min and 52 s] before and after gadolinium-based contrast agent i.v. administration; T2-weighted turbo spin-echo (TSE) sequence acquired on the three orthogonal planes [3000 ms TR, 80 ms TE, 300x256 matrix, 3 mm slice thickness with 1 mm gap, scan time of 2 min and 6 s]; a fluid attenuated inversion recovery (FLAIR) sequence acquired on the axial plane [11000 ms TR, 125 ms TE, 2800 ms TI, 320x200 matrix, 3 mm section thickness with 1 mm intersection gap, FOV 240x222x139, scan time of 5 min and 8 s] and a T2* weighted fast field echo (FFE) sequence [1039 ms TR, 16 ms TE, 35 slices with 3 mm slice thickness, gap 1 mm, 256x197 matrix, scan time of 3 min and 30 s].

### Advanced MRI techniques

Advanced techniques included DWI, DTI, MRS and PWI.

DWI sequence [3700 ms TR, 67 ms TE, 128x128 matrix, 28 slices of 4 mm thickness with 1 mm gap, b values of 0 and 1000 s/mm^2^, scan time of 44 s] was acquired in three orthogonal directions (axial, coronal, and sagittal). The resulting images were averaged in a DWI isotropic map in order to suppress effect due to anisotropy and to increase the signal to noise ratio. Firstly, an ADC isotropic map was calculated by finding the average ADC from all the available gradient directions. Then, this average ADC map was used together with the b = 0 image to create the DWI isotropic map.

Axial DTI was obtained by using a single-shot spin-echo EPI sequence [6245 ms TR, 60 ms TE, 128x128 matrix, b values of 0 and 800 s/mm^2^, 15 diffusion sensitive directions, scan time of 3 min and 58 s].

2D spectra were obtained by using a multivoxel Point-Resolved Spectroscopic Sequence (PRESS) [1700 ms TR, 144 ms TE, 19x22 acquisition matrix, 24x24 reconstruction matrix, 10 mm×10 mm×12 mm voxels size, scan time of 11 min and 57 s]. Automated optimization of gradient shimming, transmitter pulse power and water suppression were used.

Before acquiring PWI an intravenous saturation bolus injection of 3 ml gadobutrol (Gadovist^®^; Bayer Schering 1mMol/L, Berlin, Germany) was injected by means of a power injector (Medrad^®^ Spectris Solaris^®^ MR injection system) at a flow rate of 2 ml/s, followed by a 20 ml saline flush at same rate. About three minutes later a second bolus of 0.1 ml/kg b.w. contrast medium, followed by 20 ml saline flush, was i.v. automatically injected at a flow rate of 4 ml/s. A T2* weighted Fast Field Echo-Echo Planar Imaging (FFE-EPI) sequence was acquired during the second bolus passage [50 dynamic scans, 1576 ms TR, 40 ms TE, 25 slices with 4 mm slice thickness, 96x96 matrix, scan time of 1 min and 25 s].

### Image Analysis

DTI, MRS, and PWI maps were overlayed on post-contrast 3D TFE T1-weighted sequence; this permitted to place ROIs at the same anatomical level and position as respect to the lesion architecture. Post contrast 3D TFE T1 images were also used as reference to place ROIs on ADC images.

Conventional and advanced 3T MRI images were analyzed on a dedicated workstation (Extended MR WorkSpace, release 2.6.3.2; Philips Medical Systems, Best, Netherlands) in joint session by two experienced neuro-radiologists, with respectively 10 and 15 years of advanced MR imaging training, who manually placed regions of interest (ROIs) in 4 areas: Ring Enhancement (RE), Internal Cavity (IC), Peri-Lesional edema (PL), and Contralateral Normal Appearing White Matter (CNAWM). ROIs dimensions were guided by each area size and ranged from 20 mm^2^ to 40 mm^2^.

Lesions diameters ranged from 25 mm to 47 mm for abscesses, from 15 mm to 37 mm for GBMs, and from 12 mm to 42 mm for metastases.

From DSC data, blood volume maps, blood flow maps and mean transit time maps were generated and relative CBV (rCBV = CBV/CBV_cnawm_), Cerebral Blood Flow (CBF) and relative Mean Transit Time (rMTT = MTT/MTT_cnawm_) were assessed in each area.

For the analysis of MRS data, the peak heights and the peak areas at six metabolite resonances [Choline (Cho) at 3.2 ppm, Creatine (Cr) at 3.0 ppm, Succinate at 2.4 ppm, N-Acetyl Aspartate (NAA) at 2 ppm, Lactate at 1.33 ppm, and Lipids at 1.2 ppm] were estimated in voxels corresponding to IC and PE. We didn’t consider RE spectroscopic parameters for analysis because the voxel size was not small enough to allow separation between RE and IC or PE.

Cr peak area was used as internal reference for the quantification of the other metabolites.

From DWI data, ADC maps were generated and relative ADC values (rADC = ADC/ADC_cnawm_) were assessed in each area.

For analysis of DTI data, FA values were calculated in each area from the respective map.

### Statistical analysis

Significant differences between malignant lesions and benign lesions as well as between metastases and GBMs were assessed by using Mann-Withney U test.

Principal component analysis (PCA) and Partial Least Square Discriminant Analysis (PLS-DA) were performed in order to find MRI variables discriminating between malignant and benign lesions. Data were mean centered, scaled to unit variance, and log transformed before analysis.

The diagnostic accuracy of MRI biomarkers taken alone or combined by calculating the ratios between pairs of MRI biomarkers, were also investigated by using the Receiver Operating Characteristic (ROC) curve analysis. ROC curve provides the sensitivity (true positive rate) and the specificity (false positive rate) of a diagnostic test, representing the ability of a variable to discriminate between two groups; an Area Under the Curve (AUC) ≥ 0.9 indicates a very good diagnostic accuracy.

ROC curve analysis was performed by using the ROC curve analysis tool [ROC Curve Explorer and Tester (ROCCET) available at http://www.roccet.ca**]**. Softwares used for statistical analysis were SIMCA P^+^ version 11.0 (Umetrics, Umeá, Sweden), and Statistica 6.0 (Statsoft, Tulsa, OK).

## Results

**Fig 2:**
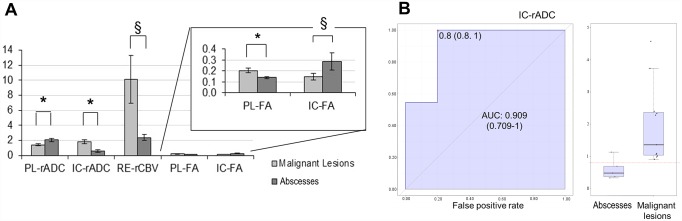
Changes of PL-rADC, IC-rADC, RE-rCBV, PL-FA, and IC-FA between abscesses vs malignant lesions (A) and ROC analysis for IC-rADC (B). Bar graphs show mean values ± standard error (SEM; n_Malignant Lesions_ = 9; n_Abscesses_ = 5). * indicates statistically significant differences as assessed by Mann-Withney U test (95% confidence level). § indicates p<0.100. The insert shows an enlarged view of the bar graph. The value of the area under curve (AUC) with the 95% confidence interval, the optimal threshold of IC-rADC, and the corresponding sensitivity and specificity are shown in the panel B.

Abscesses showed significant increase of PL-rADC value, decrease of IC-rADC value, and decrease of PL-FA value if compared to malignant masses (Table 1; Fig 2A). Furthermore, abscesses showed a trend toward decrease of RE-rCBV and increase of IC-FA respect to malignant masses without reaching statistical significance (Table 1).

**Table 1:** Mean values, standard deviations, and standard errors of the mean for PL-rADC, IC-rADC, RE-rCBV, PL-FA, and IC-FA in malignant lesions (n = 9) and abscesses (n = 5). The significance of differences (95% confidence level) was assessed by Mann-Withney U test. SD: standard deviation. SEM: standard error of the mean.

	Malignant Lesions		Abscesses		
Variable	Mean	SD (SEM)	Mean	SD (SEM)	Mann-Withney U test, p
PL-rADC	1.4	0.3 (0.1)	2.1	0.5 (0.2)	0.029
IC-rADC	1.8	0.7 (0.2)	0.6	0.3 (0.1)	0.004
RE-rCBV	10	9 (3)	2.4	0.9 (0.4)	0.083
PL-FA	0.20	0.07 (0.02)	0.14	0.02 (0.09)	0.019
IC-FA	0.15	0.09 (0.03)	0.3	0.2 (0.1)	0.089

Metastases showed significant increase of PL-rADC (1.7±0.2; Mann-Withney U test, p = 0.048) and decrease of PL-rCBV values (0.5±0.3; Mann-Withney U test, p = 0.048) as compared to GBMs (PL-rADC = 1.3±0.3; PL-rCBV = 1.3±0.7).

Multivariate data analysis such as PCA and PLS-DA did not show any discrimination between benign and malignant lesions (data not shown). Therefore, we tested the predictive power of single MRI variables or ratios between pairs of MRI variables by ROC analysis.

On the basis of our knowledge, ratios between pairs of MRI variables have not been investigated as potential biomarkers until now. However, ratios of variable pairs have been widely used in clinical chemistry and medicine and have been often proved to be better indicators for physiological or pathological conditions [27, 28]. This technique has been favoured in metabolomic studies employing a hypothesis-free approach for bulk analysis of all possible combinations of molecule pairs [29, 30]. Furthermore, it has been hypothesized that metabolite ratios may better describe individual metabolic status as compared to absolute concentrations of individual metabolites alone, since they inherently correct for individual variations of metabolite levels [31]. Therefore we chose this approach as the more efficient to identify potential MRI biomarkers of malignant lesions.

ROC curve analysis showed that IC-FA, RE-rCBV, PL-rADC, and PL-FA were poor predictor biomarkers for the differentiation between benign and malignant ring enhancing lesions (AUCs did not significantly differ from 0.5 for p>0.050) (Table 2). Conversely, IC-rADC proved to be an effective biomarker of abscesses (AUC of 0.909), and a cut-off value of 0.85 for IC-rADC provided a sensitivity of 1 and a specificity of 0.8 (Fig 2B).

**Table 2:** Receiver operating characteristic (ROC) analysis of individual MRI variables. Significance of the area under curve (AUC) was determined by the Student’s t-test taking as the null hypothesis that AUC = 0.500. IC-rADC is the only potential classifier with an AUC significantly greater then 0.500 (95% confidence level).

Variable	AUC	t-test, p
IC-rADC	0.909 (0.822–1)	0.002
PL-FA	0.889 (0.667–1)	0.052
PL-rADC	0.867 (0.699–1)	0.065
RE-rCBV	0.800 (0.566–1)	0.11
IC-FA	0.789 (0.466–0.978)	0.070

When we tested the predictor ability of combined MRI biomarkers, IC-rADC/PL-NAA, IC-rADC/IC-FA, RE-rCBV/RE-FA, IC-rADC/RE-FA, IC-rADC/PL-FA achieved higher AUCs compared to IC-rADC (Table 3).

**Table 3:** Receiver Operating Characteristic (ROC) analysis of significant combined MRI biomarkers calculated as ratios between pairs of single variable. The calculated Area Under the Curve (AUC), p values from the Student’s t-test, and fold changes calculated for the 5 best predictors are shown. The optimal cut-off point selected for the classification of malignant lesions and abscesses and the corresponding sensitivity and specificity are also shown.

Ratio	AUC	t-test, p	Fold Change	Cut-off	Specificity	Sensitivity
IC-rADC/PL-NAA	1 (1–1)	0.008	-3.025	0.359	1	1
IC-rADC/IC-FA	0.978 (0.867–1)	0.001	-2.401	2.62	1	0.9
RE-rCBV/RE-FA	0.933 (0.733–1)	0.011	-2.022	4	1	0.9
IC-rADC/RE-FA	0.911 (0.667–1)	<0.001	-2.197	2.11	0.8	1
IC-rADC/PL-FA	0.911 (0.711–1)	0.013	-1.241	2.07	0.8	0.9

The best predictor was the ratio IC-rADC/PL-NAA that provided the maximum AUC value (AUC = 1) and sensitivity and specificity of 1 with optimal cut-off value of 0.359 (Table 3).

## Discussion

PL-rADC, IC-rADC, and PL-FA values were significantly different in the two groups (benign vs malignant), but only IC-rADC reached significant diagnostic accuracy.

The usefulness of ADC in the characterization of brain abscesses is well known in literature [32] and a recent meta-analysis has confirmed that DWI has high sensitivity and specificity in the differentiation between benign and malignant ring-enhancing brain masses [16]. It is assumed that the diffusivity of water molecules in pyogenic brain abscesses cavity is restricted due to the high viscosity of pus, which contains inflammatory cells, bacteria, necrotic tissue and exudate [33–38]. Conversely, necrotic brain tumors usually show an increase of the diffusivity in their central cavity due to the hydrophilic properties of the necrosis, which is rich in cellular debris, serum, and haemorrhagic fluid [39, 40]. Consequently, the ADC values in cystic and necrotic brain tumours can be 4 to 10 times higher than in abscesses [41].

However, it should be stressed that the rate of false positives is not negligible even when the most effective IC-rADC cut-off value is taken into consideration at ROC curve analysis.

IC-rADC values showed a quite large variability with values ranging from 0.3 to 1.1 for the abscesses and from 1.0 to 3.2 for malignant lesions. This wide range may be related to different age of the abscesses at the time of the MRI exam, or related to the degree of host immune response [42–44].

Concerning IC-rADC changes, conflicting results have been reported in literature [45, 46]. Reddy and colleagues [47] observed ADC values similar to that of normal brain in 4 out of 95 brain abscesses. These findings were addressed to partial volume averaging artifacts or to intrinsic differences in the properties of diffusion in small abscesses. Park and colleagues [41] described two cases of cystic brain metastasis with central restricted diffusion, mimicking a pyogenic abscess. Chang and colleagues [48] reported a ring-enhanced fibrillary low-grade astrocytoma with a restricted diffusion content proven to be a creamy necrotic material similar to pus.

Except IC-rADC, in our study neither biomarkers taken alone nor PCA and PLS-DA models of MRI variables achieved significant diagnostic accuracy in differentiating between benign and malignant ring enhancing lesions.

In contrast, by combining each MRI biomarker with others, several ratios between pairs of single variables reached significant diagnostic accuracy with AUC values higher than those achieved by IC-rADC.

Combined biomarkers showing excellent diagnostic accuracy (AUC>0.9) were IC-rADC/PL-NAA, IC-rADC/IC-FA, RE-rCBV/RE-FA, IC-rADC/RE-FA, and IC-rADC/PL-FA.

In particular, a cut off value of 0.359 for the ratio IC-rADC/PL-NAA ensured a 100% sensitivity and a 100% specificity with a remarkable increase of diagnostic accuracy respect to IC-rADC taken alone. NAA is a sensitive biomarker for neurons integrity and its reduction has been related to different pathologic conditions. Recently, Price and colleagues [49] have reported a significant reduction of NAA/Cr levels in the invasive margins of GBMs. The increased diagnostic accuracy of IC-rADC/PL-NAA ratio, as compared to IC-rADC taken alone, can be attributed to the high predictive power of PL-NAA detected in malignant lesions and reflects their infiltrative nature.

When IC-rADC was combined with IC-FA, its specificity improved from 80% to 100%. Previous studies showed higher IC-FA values in abscesses if compared with GBMs and metastases [17, 19]. It was hypothesized that the higher IC-FA values of abscesses could be due to the presence of “orientated” inflammatory cells causing anisotropic diffusion in abscesses internal cavity. Primary and secondary brain tumours internal cavity consists of tissue debris and few inflammatory cells and so diffusion is isotropic [17, 19].

Conversely, diagnostic accuracy of IC-rADC was only slightly increased when combined to PL-FA or RE-FA (AUC = 0.911 vs AUC = 0.909).

RE-rCBV and RE-FA median values taken alone were not significantly different between the two groups. Erdogan et al have previously reported that perfusion MRI may allow the differentiation of pyogenic brain abscess from cystic brain tumors and show that RE-rCBV was higher in GBMs compared to abscesses [20]. Partially in agreement with the Erdogan study, we found that RE-rCBV shows a trend toward increase in malignant lesions compared with benign lesions. It is well known that rCBV values strongly depend on data acquisition and post-processing methods, with a large interpatient and interstudy variability, for the same tissue type [50]. Although we follow literature recommendations [51] in both acquisition and post-processing of data (reduction of the leakage effect by administering a loading dose of contrast agent prior to acquiring PWI and normalization of CBV values to the CBV values in normal-appearing white matter), the large inter-individual variability of RE-rCBV in malignant lesions probably affected the significance of our results.

However, when RE-rCBV and RE-FA were combined, the corresponding ratio was significantly different between the two groups and a cut-off value of 4 provided a 100% specificity and a 90% sensitivity. A probable reason may be that ratios correct for experimental factors that are still not well defined and for inter-individual variability.

Previous studies reported the potential of PWI in differentiating abscesses from GBMs and metastases [52, 53]. It is well known that microvascular blood volume is elevated in malignant tumors with a clear association between increased tumor neovascularity and malignancy [52, 53].

In one previous study, comparing abscesses with GBMs and metastases, FA values resulted higher in the enhancing rim of the abscess than in the enhancing rim of GBMs and metastases. Authors argued that the high FA values in the abscess rim were predominantly due to increased planar tensor values, which may be secondary to the presence of concentric layers of collagen fibers, and numerous inflammatory cells [54].

Advanced MRI techniques allow a non-invasive, quantitative method for investigating normal and pathological tissues. Thanks to the greater signal to noise ratio, 3T MRI provides more reliable and more reproducible data than 1.5T MRI [55]. Data obtained by advanced MRI techniques are considered as “potential” biomarkers for the assessment of microstructural and microvascular changes induced by brain lesions.

So far, researchers focused on identifying the best biomarker and the best cut-off value able to differentiate between different etiologies. But, it is becoming increasingly clear that the nature of a lesion cannot be fully described by a single biomarker that, inevitably, gives information about just one of the many aspects of a lesion.

Hence in this study, we started from the idea that the microstructural heterogeneity of a lesion can be described in a more comprehensive way by combining information from different MRI biomarkers, as already done in other medical research fields [22, 23, 24, 28, 29]. To this end, we proposed to apply this approach to advanced MRI techniques.

A concern of the study could be that advanced MRI data were detected in joint session by two readers [56, 57]. This modality was not performed to provide for a clinical interpretation, but, in order to reduce scattering of data and to get more reliable results, an agreement among the two readers was reached concerning the ROIs placement on MRI images.

Another limitation of the study could be that residual involuntary bias in the inclusion process cannot be excluded considering that the readers were also the ones who collected the data. However, all data were anonymized and the task of the readers was not to establish a second diagnostic judgment but only to collect the MR signal in standardized ROIs.

Although the major limitation of the study is the small sample size of the study groups and a higher statistical power is desirable, a trade off with the number of patients, that may eventually be recruited, should always be considered. We point out that the smallest measured difference between the two groups was large enough to statistically validate the sample size, as regard a pilot study [58], which can be of interest for further investigation in larger cohorts.

Strengths of the study included restrictive inclusion criteria (patients affected by lesions indistinguishable at conventional MRI undergoing same 3T MRI advanced techniques scan protocol), clinically well-defined patient groups and combination of multiple imaging biomarkers.

Aiming to improve the diagnostic accuracy, to better predict the prognosis and to guide the treatment, this study suggests that combination of multiple 3T MRI biomarkers can be more reliable than single biomarkers evaluation in the differentiation between benign and malignant single ring enhancing brain masses.

Despite the limited sample size does not allow definitive conclusion and further studies in larger cohort are needed, we point out the feasibility of the method and the novelty of this approach in the field of diagnostic neuroimaging.
